# Characteristics associated with attitudes and behaviors towards mask wearing during the COVID-19 pandemic: The Trojan Pandemic Response Initiative

**DOI:** 10.1186/s12889-023-16915-x

**Published:** 2023-10-11

**Authors:** Michele Nicolo, Eric Kawaguchi, Angie Ghanem-Uzqueda, Daniel Soto, Sohini Deva, Kush Shanker, Ryan Lee, Frank Gilliland, Jeffrey D. Klausner, Lourdes Baezconde-Garbanati, Andrea Kovacs, Sarah Van Orman, Howard Hu, Jennifer B. Unger

**Affiliations:** 1 Department of Nutrition and Food Science, California State University Los Angeles, Los Angeles, CA USA; 2 Department of Population and Public Health Sciences, Keck School of Medicine, University of Southern California, Los Angeles, CA USA; 3 Family Medicine, Keck Medicine of USC, Los Angeles, CA USA; 4 Keck School Medicine of USC, University of Southern California, Los Angeles, USA

**Keywords:** SARS-COV-2, COVID-19, University sample, Face mask, Health behavior

## Abstract

**Background:**

Attitudes and behaviors towards mask wearing may influence the ability to reduce transmission of COVID-19 and other diseases.

**Methods:**

University students, staff, and faculty (N = 9653) responded to an email invitation to complete electronic surveys (November 2021 and April 2022). Surveys included 19 items measuring attitudes and behaviors towards mask wearing from the Understanding America Study. Linear mixed models including variables for sex, age group, division, race and ethnicity, political affiliation, and history of COVID-19, were used to estimate the mean difference of the mean score for attitudes and behavior between Time 1 (November 2021) and Time 2 (April 2022).

**Results:**

Participants were mostly female (62.1%), students (70.6%), White (39.5%) and Asian (34.7%). More than half identified their political affiliation as Democrat (65.5%). Characteristic variable-by-time interactions for difference in mean mask attitude scores difference were significant at Time 1 (T1) and Time 2 (T2) between Black and White participants (B = 0.18 (0.05), 95% CI: 0.07, 0.28, p = 0.001), Asian and White participants (B = 0.07 (0.02), 95% CI: 0.03–0.12, p = 0.001), participants with self-reported history of COVID-19 and no history of COVID-19 (B= -0.13 (0.02), 95% CI: -0.07, -0.18, p < 0.0001), females and males (B = 0.07 (0.02), 95% CI: 0.03, 0.11, p = 0.001), Republicans and Democrats (B= -0.18 (0.04), 95%CI: -0.26, -0.10, p < 0.0001) and Independents and Democrats (B= -0.10 (0.03), 95%CI: -0.15, -0.05, p < 0.0001). Mean difference in mean scores for mask behaviors at Time and Time 2 were significant between participants with COVID-19 and participants who did not have COVID-19 (B= -0.12 (0.04), 95% CI: -0.19, -0.04, p = 0.004), students compared to faculty and staff (B=-0.22 (0.05), -0.32, -0.12, p < 0.0001), between Republicans and Democrats (B-= -0.16 (0.07), 95% CI: -0.28, -0.03, p = 0.020, and between Independents and Democrats (B=-0.08 (0.04), 95% CI: -0.16, -0.002, p = 0.04).

**Conclusion:**

Race and ethnicity, political affiliation, and division may affect attitudes and behaviors in mask wearing. Further investigation into how characteristics influence public health measures such as mask wearing is needed to contain the spread of the COVID-19 virus, other infectious diseases, and future pandemics.

## Introduction

Wearing a face mask is used as a prevention against the spread of infections, and most recently encouraged as primary prevention practice against the spread of COVID-19 [1–4]. Published research results suggest face mask use can greatly reduce the risk of respiratory virus transmission including COVID-19 by > 60% [5, 6]. The recommendation for face masks is intended to mitigate the spread of transmission particularly among those who are infected but asymptomatic. The benefit of face masks is dependent on consistent and proper use of masks covering both the nose and the mouth [6, 7]. Despite the findings showing mask wearing as an effective practice against the spread of COVID-19, mask wearing has become a controversial and political topic [8–11].

In some instances, mask wearing has become less about public health practice and more about individual preferences influenced by many characteristics [12, 13]. Differences in mask wearing among age groups have been observed with younger adults less likely to wear masks compared to older adults [8]. Lower perceived risk of COVID-19 among younger adults may have influenced mask behaviors as older adults are considered more at risk for severe disease [14, 15]. Given the rapid developments surrounding the COVID-19 pandemic, few studies have measured changes in attitudes and behaviors towards mask wearing especially among age groups [16, 17].

Other characteristics associated with mask wearing attitudes and behaviors include race and ethnicity. Lower rates of face mask use have been observed among Black and Hispanic adults, compared to non-Hispanic whites, earlier during the pandemic in 2020 [4]. This coincided with greater rates of COVID-19 infections among these groups, who are also at greater risk for health disparities and low vaccine uptake [18–20]. Mask wearing has become a political talking point particularly within the United States [21]. Misinformation circulated through various media sources has influenced how individuals view the science behind mask wearing as a prevention measure [22]. Political affiliation is associated with attitudes and behaviors towards wearing masks [23] and certain political ideologies have been linked with greater mistrust and disbelief surrounding mask wearing [24].

Most studies investigating attitudes and behaviors towards mask wearing occurred earlier during the pandemic before vaccines were available [25–29] when the focus was on mitigation behaviors. Despite widespread availability of the COVID-19 vaccine and related outreach, at the time of this study only 67.8% of the total U.S. population are fully vaccinated (received 2 doses of the vaccine) and 51.8% of adults 18 years and older have received their first booster dose [30]. As of September 2022, 35.5% of adults 50 years and older have received their second booster vaccine [30], and as of July 2023, the CDC reports 79.1% of adults 18 years and older have completed the primary vaccination series (received two vaccinations) and only 20.5% have received an updated bivalent booster vaccination [31]. Most recent data on effectiveness of a second COVID-19 booster vaccination 15 weeks after administration is only 43.7% effective against symptoms and 56.5% against COVID-19 related hospitalizations [32]. In May of 2023, the U.S. government declared an end to the public health emergency response related to COVID-19 pandemic [33]. This position drastically reduced public health surveillance testing with the exception of hospitalization and associated mortality data, despite COVID-19 continuing to be a concern among certain communities [33, 34]. Currently there are no guidelines regarding COVID-19 vaccination recommendations beyond the second booster vaccine [33]. The COVID-19 pandemic has brought the ability and capacity of the public health to manage future pandemics to the forefront. The readiness and acceptance of the population to re-implementation health behavior measures such as mask wearing in the event of a COVID-19 resurgence or future pandemic is uncertain. Most studies investigating attitudes and behaviors towards mask wearing occurred earlier during the pandemic before vaccines were available, and there was a focus on mitigation behaviors. Despite widespread availability of the COVID-19 vaccine and related outreach, only 67.8% of the total U.S. population are fully vaccinated (received 2 doses of the vaccine), and 51.8% of adults 18 years and older have received their first booster dose and 35.5% of adults 50 years and older have received their second booster vaccine as of September 2022 [30]. Because a significant proportion of the U.S. population have yet to be fully vaccinated and vaccine immunity wanes over time, continued use of public health practices including mask wearing may be warranted; however, mask mandates and recommendations have declined. How the change in mask wearing policies have influenced attitudes and behaviors despite the continued spread of COVID-19 is unknown.

In a previous study, differences in self-reported intention to receive a COVID-19 vaccine among staff and faculty at a large, diverse university in Los Angeles were observed [23]. Results from this study suggest having a political affiliation to a party other Democrat was associated with a greater likelihood of not receiving a COVID-19 vaccine [23]. In the present study, we aim to describe characteristics associated with changes in mask wearing attitudes and behaviors among students, faculty, and staff within a large, diverse university between November 2021 and April 2022. We will investigate variation in mask wearing attitudes and behaviors among racial and ethnicity groups. We anticipate stronger positive mask attitudes and behaviors among older adults compared to younger adults, and therefore greater positive attitudes and behaviors among staff and faculty compared to students. Furthermore, we expect more negative attitude and behavior towards mask wearing among individuals identifying with a political affiliation other than Democrat and among races and ethnicities other than White.

## Methods

### Participants

Participants were students, faculty, and staff at a large university located in Southern California. Participants were eligible if they were currently enrolled as a student or worked as staff or faculty and were at least 18 years of age and provided informed consent.

### Procedure

This study has been approved by the university Institutional Review Board. Methods and procedures have been published elsewhere [23]. Briefly, all students enrolled at the university in addition to staff and faculty with a university affiliated email were invited by email to participate in a brief COVID-19 survey. Participants who completed the first wave of the study were subsequently invited to continue in the study and compete three additional surveys (waves) approximately 3–6 months apart. Survey responses were collected from 11/29/2021 to 4/22/2022. Questions pertaining to the outcome of interest for this study were included in waves 2 (Time 1) and 3 (Time 2) surveys; therefore, only responses for Time 1 and Time 2 were included in the analyses. To assess directional change in scores, only participants completing surveys at both times were included.

### Outcome variable

Attitude toward masks scores were calculated using responses to 10 survey questions from the ” Understanding America Study” [35]. Examples of statements include: “Wearing a mask is not needed because I am not infected”, “Wearing a mask is not needed when I am with other people who are healthy”, and “Wearing a mask is unnecessary because coronavirus is not a serious threat to people like me. “(Cronbach alpha = 0.82). Responses ranged from “strongly disagree” to “strongly agree”. Responses were numerically coded with some responses recoded so higher scores reflect more positive attitudes towards masks. Mean scores were calculated for each time.

Mask behavior scores were calculated using participant responses to frequency of mask use in different scenarios and situations. Participants were asked “In the past week, when do you wear a mask?” followed by several scenarios including religious functions, general errands and at work. Responses ranged from “never” to “always”. (Cronbach alpha = 0.75). Reponses were numerically coded with higher scores reflecting greater mask behaviors. Mean mask behavior scores were calculated for each time.

### Independent variables

Demographic variables included self-reported sex (male, female), race/ethnicity (Black, Asian, Latinx, Other races and ethnicities and White), division (student or staff and faculty) and age. Greater than 80% of students were between ages 18 and 24 years, and ages among staff and faculty ranged between 18 and 85 years. Political affiliation was categorized as Republican, Democrat, Independent or something else, and self-reported history of COVID-19 was categorized as “yes” or “no”.

### Data analysis

Continuous variables were reported as mean and standard deviation (SD), and categorical variables as a percentage. Linear mixed models (LMM) were used to examine the association of the difference in mean score difference in attitudes and behaviors toward mask use at Time 1 and 2, and a set of time-constant variables (age, sex, division, race and ethnicity, political affiliation, and self-reported COVID-19 status) The outcome model included a random (subject − specific) intercept.

## Results

Demographic characteristics are shown in Table 1. The sample was mostly female (62.1%), White (39.6%) or Asian (34.6%), and students (70.6%). Participants were mostly Democrats (65%) and did not have a self-reported history of COVID-19 (79.1%).

### Linear mixed model results

Results of linear mixed model analyses for differences in mean score difference for attitudes towards mask wearing by characteristic and time-interactions are shown in Table 2. At Time 1, White participants had lower attitude scores compared to Black, Asian, Latinx and Other races and ethnicities. Attitude scores were higher among Black, Asian, and Other races and ethnicities for their respective race and ethnicity at Time 2 compared to Time 1; whereas scores for Latinx and White participants were lower at Time 2 compared to Time 1. Race and ethnicity-by-time interactions suggest the difference in mean mask attitude score difference between Black and White participants at T1 and T2 are significantly different (B = 0.18 (0.05), 95% CI: 0.07, 0.28, p = 0.001). At Time 1 Black participants’ attitudes towards mask wearing were higher than White participants (5.19 vs. 5.04), and at Time 2 the difference in mean attitudes scores widened between Black and White participants (5.24 vs. 5.01).

**Table 1:** Descriptive characteristics (N=9653)

Variable	N (%)
Race and ethnicity	
Black Asian Latinx Other White	446 (4.6) 3346 (34.6) 1549 (16) 487 (5) 3825 (39.6)
Sex	
Female Male	6012 (62.1) 3671 (37.9)
Age Group (years)	
18–21 22–24 25–31 32–85	2507 (25.9) 2622 (27.1) 2165 (22.4) 2389 (24.7)
Division	
Student Staff/faculty	6838 (70.6) 2845 (29.4)
Political affiliation	
Republican Independent Something else Democrat	449 (6.6) 1495 (21.9) 449 (6.6) 4438 (65)
Had COVID-19	
No Yes	3349 (79.1) 884 (9.1)

N: number; %: percentage

**Table 2:** Mean scores and standard deviation (SD) and linear mixed model results for mean differences in attitudes towards mask wearing by characteristic and time-interactions

Variable	Mean Attitude Score Mean (SD)		Main Effects			Time Interaction Model ^b^		
N = 9653	T1	T2	B (SE)	95% CI	p	B (SE)	95% CI	p
Race and ethnicity								
Black Asian Latinx Other White ^a^	5.19 (0.71) 5.17 (0.68) 5.12 (0.72) 5.11 (0.75) 5.04 (0.73)	5.27 (0.62) 5.20 (0.70) 5.11 (0.78) 5.19 (0.80) 5.01 (0.78)	0.000 (0.55) 0.15 (0.24) 0.03 (0.03) 0.03 (0.05)	− .0.1, 0.11 0.11, 0.20 -0.02, 0.09 -0.07, 0.13	0.99 < 0.0001 0.318 0.593	0.18 (0.05) 0.07 (0.02) 0.04 (0.03) 0.15 (0.05)	0.07, 0.28 0.03, 0.12 -0.01, 0.10 0.01, 0.20	0.001 0.001 0.127 0.02
Sex								
Female Male ^a^	5.20 (0.64) 4.92 (0.82)	5.21 (0.67) 4.88 (0.87)	0.19 (0.02)	0.15, 0.23	< 0.0001	0.07 (0.02)	0.03, 0.11	0.001
Age Group (years)								
18–21 22–24 25–31 32–85 ^a^	5.03 (0.73) 5.05 (0.75) 5.09 (0.72) 5.19 (0.67)	5.02 (0.78) 5.07 (0.77) 5.11 (0.76) 5.16 (0.72)	-0.16 (0.04) -0.15 (0.04) -0.12 (0.03)	-0.25, -0.08 -0.23, -0.07 -0.19, -0.06	< 0.0001 < 0.0001 < 0.0001	-0.03 (0.04) 0.05 (0.04) 0.04 (0.03)	-0.11, 0.85 -0.03, 0.12 -0.01, 0.10	0.40 0.21 0.14
Division								
Student Faculty/staff ^a^	5.05 (0.74) 5.19 (0.67)	5.06 (0.78) 5.16 (0.72)	-0.08 (0.03)	-0.14, -0.01	0.02	0.04 (0.03)	-0.03, 0.10	0.20
Political affiliation								
Republican Independent Something else Democrat ^a^	4.46 (1.03) 4.86 (0.86) 5.06 (0.79) 5.24 (0.56)	4.34 (1.10) 4.82 (0.91) 5.08 (0.87) 5.25 (0.59)	− .67 (0.04) − .30 (0.03) − .09 (0.05)	-0.76, -0.59 -0.35, -0.25 -0.17, 0.003	< 0.0001 < 0.0001 0.06	-0.18 (0.04) -0.10 (0.03) -0.03 (0.04)	-0.26, -0.10 -0.15, -0.05 -0.11, 0.06	< 0.0001 < 0.0001 0.49
Had COVID-19								
Yes No ^a^	4.96 (0.78) 5.15 (0.69)	4.89 (0.85) 5.16 (0.72)	-0.11 (0.03)	-0.16, -0.06	< 0.0001	-0.13 (0.02)	-0.17, -0.08	< 0.0001
Time ^b^			-0.10 (0.03)	-0.15, -0.05	< 0.0001			

^a^ Reference variable

^b^ Association variables with score change over time since the start of the study

N: number; M: mean; SD: standard deviation; B: Beta (difference in mean differences between Time 1 and Time 2), adjusted estimate coefficient; SE: standard error CI: confidence interval; p: p-value

Significant results were also observed in difference in mean attitudes score difference between Asian and White participants (B = 0.07 (0.02), 95% CI: 0.03–0.12, p = 0.001) and Other races and ethnicities and White participants (p = 0.02). Asian and Other race ethnicities had greater attitude scores at Time 1 compared to White participants (5.17 and 5.11 vs. 5.04), and at Time 2 the difference in mean score difference further increased among Asians and Other races and ethnicities compared to White participants (5.22 and 5.19 vs. 5.01). No significant interaction by-time in mean mask attitudes was observed between Latinx and White participants.

The sex-by-time interaction suggests difference in mean difference for mask attitude score between females and males at T1 and T2 is significant (B = 0.07 (0.02), 95% CI: 0.03, 0.11, p = 0.001). At Time 1 females had greater attitude scores compared to males (5.20 vs. 4.92), and at Time 2 the difference in the mean scores difference further increased between females and males (5.21 vs. 4.88). The mean difference in mask attitude score difference between participants with self-reported history of COVID-19 and no history of COVID-19 at T1 and T2 are significantly different (B= -0.13 (0.02), 95% CI: -0.07, -0.18, p < 0.0001). At Time 1 participants reporting no history of COVID-19 had greater attitude scores compared to those reporting a history of COVID-19 (5.15 vs. 4.96). Scores at T2 show further widening in the difference in mean scores between participants who had COVID-19 and those reporting no history (5.16 vs. 4.89).

At T1 participants identifying their political affiliation as Democrat had higher mean attitude scores towards mask wearing compared to other political affiliations, while those identifying as Republican had the lowest mean scores in comparison. Mean attitude scores were slightly higher at T2 compared to T1 among Democrats (5.24 and 5.25). Mean score among Republicans were lower at T2 compared to T1 (4.46 and 4.34). The difference in mean attitude score difference between Republicans and Democrats at T1 and T2 are significant (B= -0.18 (0.04), 95%CI: -0.26, -0.10, p < 0.0001). Similarly, differences in mean attitude score differences between T1 and T 2 were significant among Independents compared to Democrates (B= -0.10 (0.03), 95%CI: -0.15, -0.05, p < 0.0001). Mean scores for Independents decreased between T1 (4.86) and T2 [(4.82) (p < 0.0001)]. Interactions between T1 and T2 among age groups and division were not significant.

Mean scores towards mask wearing behavior are shown in Table 3. Among races and ethnicities, White participants had lowest mean behavior scores compared to all others. While mean behavior scores were lower among all characteristics between at T2 compared to T1, participants within the youngest age quartile (18 to 21 years), students, participants identifying as Republican and those reporting having a history of COVID-19 had the lowest scores within their respective categories at both T1 and T2. Differences in mean behavior score difference at T1 and T2 are significant between participants who self-reported having COVID-19 and those who did not have COVID-19 (B= -0.12 (0.04), 95% CI: -0.19, -0.04, p = 0.004). A greater difference in mean behavior scores for participants reporting a history of COVID-19 was observed at T2 (2.75) compared to T1 (2.98) scores. Mean scores for those reporting no history decreased to a lesser extent at T2 (2.97) compared to T1 (3.14). A significant difference in the mean behavior score difference among faculty, staff, and students at T1and T2 were also observed (B=-0.22 (0.05), -0.32, -0.12, p < 0.0001). The differences in the mean score difference widened further at T 2 between faculty and staff and students. Mean mask behavior scores decreased among all political affiliations at T2. Significant differences in mean behavior scores difference at T1and T2 were observed among Republicans (2.98 and 2.72) compared to Democrats [(3.11 and 2.95); B-= -0.16 (0.07), 95% CI: -0.28, -0.03, p= -0.02)], and Independents (3.13 and 2.87) compared to Democrats (B=-0.08 (0.04, 95% CI: -0.16, -0.002, p = 0.04). Significant interactions between race and ethnicity, sex and age groups were not observed.

**Table 3:** Mean scores and standard deviation (SD) and linear mixed model results for mean differences in behaviors towards mask wearing by characteristic and time-interactions

Variable	Mean Behavior Score Mean (SD		Main Effects			Time Interaction Model ^b^		
N = 9653	T1	T2	B (SE)	CI	p	B (SE)	CI	p
Race and ethnicity								
Black Asian Latinx Other White ^a^	3.39 (1.07) 3.39 (0.99) 3.35 (0.99) 3.23 (1.02) 2.74 (0.91)	3.19 (1.04) 3.18 (0.95) 3.16 (1.00) 3.06 (1.01) 2.59 (0.88)	0.64 (0.08) 0.69 (0.03) 0.62 (0.04) 0.49 (0.07)	0.48, 0.80 0.63, 0.76 0.54, 0.70 0.36, 0.64	< 0.0001 < 0.0001 < 0.0001 < 0.0001	-0.08 (0.09) -0.01 (0.04) -0.08 (0.04) -0.03 (0.08)	-0.25, 0.09 -0.08, 0.06 -0.17, 0.01 -0.18, 0.13	0.35 0.82 0.07 0.73
Sex								
Female Male ^a^	3.11 (0.98) 3.09 (1.05)	2.93 (0.95) 2.89 (1.03)	0.01 (0.03)	-0.05, 0.01	0.75	0.02 (0.03)	-0.04, 0.09	0.50
Age Group (years)								
18–21 22–24 25–31 32–85 ^a^	2.94 (1.03) 3.08 (1.02) 3.07 (0.97) 3.23 (0.99)	2.68 (0.98) 2.82 (0.95) 2.93 (0.96) 3.09 (0.98)	-0.27 (0.06) -0.15 (0.06) -0.24 (0.05)	-0.29, -0.15 -0.27, -0.05 -0.32, -0.15	< 0.0001 0.01 < 0.0001	-0.05 (0.07) 0.04 (0.06) 0.10 (0.05)	-0.17, 0.08 -0.08, 0.16 0.001, 0.19	0.48 0.54 0.05
Division								
Student Staff/faculty ^a^	3.02 (1.01) 3.22 (0.98)	2.78 (0.96) 3.10 (0.97)	-0.01 (0.05)	-0.10, 0.09	0.75	-0.22 (0.05)	-0.32, -0.12	< 0.0001
Political affiliation								
Republican Independent Something else Democrat ^a^	2.98 (1.16) 3.13 (1.10) 3.11 (1.05) 3.11 (0.96)	2.72 (1.15) 2.87 (1.05) 2.93 (0.98) 2.95 (0.94)	-0.09 (0.06) 0.04 (0.04) 0.02 (0.06)	-0.21, 0.03 -0.04, 0.11 -0.10, 0.15	0.13 0.34 0.74	-0.16 (0.07) -0.08 (0.04) 0.07 (0.07)	-0.28, -0.03 -0.16, -0.002 -0.07, 3.09	0.02 0.04 0.35
Had COVID-19								
No Yes	3.14 (0.99) 2.98 (1.04)	2.97 (0.96) 2.75 (1.04)	-0.06 (0.04)	-0.14, 0.01	0.08	-0.12 (0.04)	-0.19, -0.04	0.004
Time ^b^			-0.16 (0.05)	-0.23, -0.08	< 0.0001			

^a^ Reference variable

^b^ Association variables with score change over time since the start of the study

N: number; SD: standard deviation; B: Beta (difference in mean differences between Time 1 and Time 2), adjusted estimate coefficient; SE: standard error CI: confidence interval; p: p-value

## Discussion

The results of this study showed attitudes and behaviors towards mask wearing differed among subgroups of students, faculty, and staff within a large university population. Furthermore, changes in attitude and behavior scores over time were observed based on race and ethnicity, political affiliation, COVID-19 infection status, and division. While we had anticipated differences in attitude and behavior scores among political affiliations, the results of this study provided further insight into important differences based on race and ethnicity and age groups.

Results from previous studies have identified disparities in COVID-19 infection rates and mitigation practices among Black communities in the U.S [4, 36, 37]. These disparities stem from mistrust of scientific and government entities over a long-standing history of racial disparities among underserved communities. Because of this we had anticipated lower mask attitude and behavior scores among certain races and ethnicities particularly among Black students, faculty, and staff participants; however, we observed Black participants not only had higher mask attitude scores compared to White participants, but their scores also increased over time suggesting attitudes towards mask wearing improved. Similarly, Black participants had higher mean mask behavior scores at both times points than White participants. Although university masking requirements changed during the time of data collection making mask wearing optional, mean scores among Black participants decreased from T1 to T2, but remained higher than White participants,

Attitudes and behaviors towards mask wearing has become a form of political expression [38, 39]. Among certain groups and political affiliations, mask wearing may not be viewed in the context of reducing virus transmission but as a sign of government infringement on civil liberties [38]. In a previous study using a subsample of the same cohort [38], we observed differences in COVID-19 vaccine intention based on political affiliation [23]. We observed participants identifying as Republican or Independent self-reported they were less likely to receive a COVID-19 vaccine [23]. Considering these findings, we expected to find differences in mask attitude and behavior scores to align according to political affiliation. We observed participants identifying with a political affiliation other than Democrat had lower mask attitude scores at both T1 and T2 and those with Republican affiliation having the lowest mean mask attitude score. Mean mask behavior scores at T1 and T2 followed a similar pattern for Republican and Independent affiliations. We observed a lower mask behavior score among all political affiliations at T2; however, Democrat mask behavior scores remained the highest, whereas Republican affiliation were the lowest in the category at both times. Our findings are consistent with other published studies observing differences in COVID-19 prevention practices among political affiliations [39, 40].

This study is unique in describing differences among students, staff, and faculty towards adoption and adherence to using masks. Returning to in-person learning on campus relied heavily on implementing mitigation measures such as mask wearing. The difference in mean behavior scores between students and faculty/staff suggest the messaging on necessary public health measures needs to be specific for students. At both time points, students’ mean behavior scores were lower compared to faculty and staff and trended towards the more negative range. The university policy on wearing masks while in-person on campus was consistent regardless of division; however, delivery and dissemination on need for mask wearing did not influence this group. Although university masking requirements may have eased during the time of this study, faculty and staff likely continued with this behavior compared to students. COVID-19 vaccination was required for all students, faculty, and staff as of Fall 2021 semester. We did not investigate whether vaccination may have played a role influencing mask wearing behaviors.

This study is the first to investigate changes in attitudes and behaviors of mask wearing during a dynamically evolving pandemic. This study included a large, diverse sample of students, faculty and staff within Los Angeles, California which is one of the most ethnically diverse cities in the U.S. The longitudinal aspects of our study provided insight into attitudes and behaviors at two different time points. This is particularly important as aspects of the COVID-19 pandemic have rapidly changed since first identified as a global pandemic, and COVID-19 continues to be a public health concern despite no longer being considered a public health emergency. While we are not certain of a COVID-19 resurgence, COVID-19 continues to be prevalent. This study provides additional information on the acceptance and practice of health behaviors in the event of similar public health emergency or pandemic. Furthermore, this study suggests demographic characteristics need to be considered when implementing public health measures.

### Limitations

The data included in this study are primarily self-reported and therefore subject to bias. We did not include data regarding COVID-19 vaccination status in our analyses which may have influenced mask wearing behaviors. Furthermore, we did not account for specific university policy changes in mask wearing as these changes may have also influenced mask behaviors. International student status was not included in the analyses due to the lack of self-reported data. These results are based on a highly diverse population; however, participants were primarily White and Asian, students, identified as female and Democrat, therefore, results cannot be generalized to other populations.

## Conclusions

Attitudes and behaviors towards mask wearing among university students, staff, and faculty differ by race and ethnicity, political affiliation, and university division. COVID-19 continues to be a public health concern especially among those who are unvaccinated and individuals who have not received a bivalent booster dose. Although no longer mandated, mask wearing continues as a measure to reduce the risk of COVID-19 infection. This study identifies characteristics that may influence the acceptance and adoption of health behaviors and policies especially in a university setting in the event of a COVID-19 resurgence, the need to protect against infectious diseases like the flu, and future pandemics.

## Data Availability

The datasets used and/or analyzed during the current study are available from the corresponding author on reasonable request.
